# Demonstrating Feasibility of the DIGNITY Intervention for Delivery in Rural Nursing Home Communities

**DOI:** 10.1093/geroni/igaf122.2071

**Published:** 2025-12-31

**Authors:** Erin Kitt-Lewis, Liza Behrens, Kalei Kowalchick, Caroline Madrigal, Terrence Murphy, Jennifer Kraschnewski, Kimberly Van Haitsma, Marie Boltz

**Affiliations:** The Pennsylvania State University, University Park, Pennsylvania, United States; The Pennsylvania State University, University Park, Pennsylvania, United States; VA Boston Healthcare System, Boston, Massachusetts, United States; The Pennsylvania State University, University Park, Pennsylvania, United States; The Pennsylvania State University, University Park, Pennsylvania, United States; The Pennsylvania State University, University Park, Pennsylvania, United States

## Abstract

The DIGNITY intervention is a newly co-developed risk management strategy aimed at reducing barriers and enhancing staff engagement in risk mitigation to support residents’ care and activity preferences. This comprehensive staff-focused behavioral intervention remains untested. Therefore, a pilot test was conducted to evaluate its acceptability, appropriateness, feasibility, and preliminary efficacy among direct-care nursing home (NH) staff members. A 12-week, two-arm cluster randomized controlled trial was conducted in four rural Pennsylvania NHs. Staff (N = 54) and residents (N = 57) were recruited. All staff participated in a 60-minute introductory session and received a training manual. They were asked to refer to the manual during routine care planning for enrolled residents and while participating in six bi-weekly web-based coaching sessions. Staff rated their intention to honor residents’ risky preferences, as well as the acceptability, feasibility, and appropriateness of the intervention through surveys with closed and open-ended questions. Structured interviews collected residents’ satisfaction with preference fulfillment. Staff had an 83% retention rate and an 80% survey response rate. In the DIGNITY group, average scores for acceptability (4.19), appropriateness (4.24), and feasibility (3.99) exceeded benchmarks with supporting qualitative remarks, indicating high feasibility, acceptability, and appropriateness. Positive trends were seen in staff intent to honor residents’ preferences. A t-test revealed a statistically significant difference in resident satisfaction between study arms (p = .04). The DIGNITY intervention is feasible and shows promise in supporting NH staff engagement in risk mitigation for person-centered dementia care. Future research should assess DIGNITY’s efficacy across a larger sample of NHs.

